# Diabetic neuropathy: A NRF2 disease?

**DOI:** 10.1111/1753-0407.13524

**Published:** 2023-12-29

**Authors:** Monica Neagu, Carolina Constantin, Mihaela Surcel, Adriana Munteanu, Cristian Scheau, Ilinca Savulescu‐Fiedler, Constantin Caruntu

**Affiliations:** ^1^ Immunology Department Victor Babes National Institute of Pathology Bucharest Romania; ^2^ Pathology Department Colentina Clinical Hospital Bucharest Romania; ^3^ Doctoral School, Faculty of Biology University of Bucharest Bucharest Romania; ^4^ Department of Physiology “Carol Davila” University of Medicine and Pharmacy Bucharest Romania; ^5^ Department of Internal Medicine – Coltea Clinical Hospital, ”Carol Davila” University of Medicine and Pharmacy Bucharest Romania; ^6^ Department of Dermatology “Prof. N.C. Paulescu” National Institute of Diabetes, Nutrition and Metabolic Diseases Bucharest Romania

**Keywords:** biomarker, diabetic neuropathy, inflammation, NRF2, therapy target

## Abstract

The transcription factor nuclear factor erythroid 2‐related factor 2 (NRF2) has multifarious action with its target genes having redox‐regulating functions and being involved in inflammation control, proteostasis, autophagy, and metabolic pathways. Therefore, the genes controlled by NRF2 are involved in the pathogenesis of myriad diseases, such as cardiovascular diseases, metabolic syndrome, neurodegenerative diseases, autoimmune disorders, and cancer. Amidst this large array of diseases, diabetic neuropathy (DN) occurs in half of patients diagnosed with diabetes and appears as an injury inflicted upon peripheral and autonomic nervous systems. As a complex effector factor, NRF2 has entered the spotlight during the search of new biomarkers and/or new therapy targets in DN. Due to the growing attention for NRF2 as a modulating factor in several diseases, including DN, this paper aims to update the recently discovered regulatory pathways of NRF2 in oxidative stress, inflammation and immunity. It presents the animal models that further facilitated the human studies in regard to NRF2 modulation and the possibilities of using NRF2 as DN biomarker and/or as target therapy.

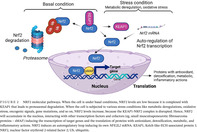

## Graphical abstract

Highlights

- Nuclear factor erythroid 2‐related factor 2 (NRF2) is an oxidant stress response transcription factor, and is involved in metabolism, inflammation, autophagy, proteostasis, mitochondrial physiology, and immune responses.
- Chronic hyperglycemia in diabetes and prediabetes conditions induces a decrease in NRF2 that leads to an increase of oxidative stress and inflammation.
- NRF2 links neuroinflammation and apoptotic pathways and contributes to the development of diabetic neuropathy.

## 1 INTRODUCTION

The transcription factor nuclear factor erythroid 2‐related factor 2 (NRF2) is involved in myriad cellular processes, from oxidative stress to protein degradation. Due to its structure, it interacts with various molecules to induce these complex cellular functions.

NRF2, belongs to the subfamily of basic leucine zipper (bZIP) transcription factors along with other members such as nuclear factor erythroid‐derived 2 (NFE2) and NRF1, NRF2, and NRF3. It is encoded by the gene nuclear factor, erythroid 2 like 2 (NFE2L2).^1^ NRF2 has several NRF2‐ECH homology (Neh) domains that control the NRF2 transcriptional activity. The bZip within Neh1 domain heterodimerizes with sMAF (small musculoaponeurotic fibrosarcoma proteins K, G, F) and other bZip proteins in order to recognize antioxidant response elements (ARE) inducing a subsequent activation of gene transcription. The Neh2 domain interacts with the Kelch domain from Kelch‐like‐ECH‐associated protein 1 (KEAP1) mediating NRF2 ubiquitination and degradation.^2^ The group of Neh3‐5 domains are transcriptional activation structures by binding to transcriptional machinery.^3^ The Neh6 domain has two redox‐independent degrons (DSGIS and DSAPGS) that bind to E3 ubiquitin ligase β‐transducin repeat‐containing protein, which, in oxidatively stressed cells, mediates NRF2 degradation.^4^ The Neh7 domain interacts with retinoic X receptor alpha, repressing NRF2 activity.^5^

All the described domains modulate NRF2 activity on target genes at transcriptional and posttranscriptional levels. Studies published in 2020 have shown that there are new NRF2 target genes beyond its already known redox‐regulating capacities. Therefore, genes that are involved in inflammation regulation, proteostasis, unfolded protein response, autophagy, and metabolism were recently reported.^6^

As there are many diseases that have oxidative stress‐related pathological background, NRF2‐target genes are essential in various diseases such as cardiovascular diseases, metabolic syndrome, neuronal degeneration, autoimmune disorders, and cancer.^7^, ^8^, ^9^ In various recent animal models, many additional ARE containing genes were found (eg, Nfe2l2‐knockout mice,^10^ Keap1‐knockdown mice, Keap1‐knockout mice,^11^ constitutive active NRF2‐E79Q‐knockin mice^12^) alongside the already mentioned oxidative stress target genes.

The International Diabetes Federation reported in 2019 that over 400 million people worldwide are diagnosed with diabetes,^13^ distributed as over 100 million individuals in China, 73 million in India, and 30 million in the United States.^14^ The same number of individuals are diagnosed with prediabetes^15^, ^16^, ^17^ and the health care system is also burdened by the comorbidities developed by patients with diabetes.^18^ Amid the complications triggered by diabetes, the damage inflicted upon peripheral and autonomic nervous systems triggers various forms of neuropathy, and the incidence of this complication is not low as it will occur in half of patients diagnosed with diabetes.^19^, ^20^

Diabetic neuropathy (DN) affects both central (CNS) and peripheral nervous systems and inflicts deregulations at both levels.^21^ Furthermore, central DN has Alzheimer's disease‐like characteristics increasing the risk of dementia,^22^ comorbid depression, and anxiety^23^ and results from oxytocinergic system deregulation.^24^ One of the mechanisms involved in the development of DN is impaired insulin support.^25^ Increased glucose leads to increased glutamate levels, which is an excitatory neurotransmitter that induces cognitive dysfunction and CNS damage.^26^ Hyperglycemia damages the structure and function of the blood–brain barrier (BBB), affecting CNS.^27^ As the BBB is the essential physiological barrier in drug delivery^28^ the only drug that is used in DN is epalrestat. This is an aldose reductase inhibitor with proven safety and efficacy.^29^ This lack of efficacious medication strengthens the need to seek out further therapy targets in DN, such as Nrf2.

The hyperglycemic conditions generated in diabetes associate mitochondrial deregulations, an increase in the reactive oxygen species (ROS) production and deregulations in the antioxidant defense system.^30^ The exacerbated generation of ROS inflicts damages to the peripheral nerves^31^ while the chronic hyperglycemic status deregulates mitochondrial functions, triggering injuries in the dorsal root ganglia and axons of sensory neurons, while reducing motor/sensory conduction speed.^32^, ^33^

The pathophysiology of DN involves complex inflammatory and apoptotic processes.^34^, ^35^, ^36^ Due to the fact that human papillomavirus (HPV) induces an ubiquitary infection causing profound chronic inflammatory processes,^37^ HPV infections were recently studied in DN, as histologically, diabetic foot alterations are similar with the lesions induced by HPV^38^

Within these intricate inflammatory mechanisms, NRF2 bridges inflammatory and apoptotic pathways and regulates antioxidant proteins (eg, detoxifying enzymes) through the ARE binding site. Moreover, NRF2 links neuroinflammation and apoptotic pathways affecting the progression of DN.^39^ The hyperglycemic status developed in diabetes stimulates oxidative stress and inflammation, downregulating the activity of NRF2. Acute hyperglycemia intensifies the expression of NRF2, whereas chronic hyperglycemia decreases it. The NRF2 downregulation induces microvascular changes, leading to DN.^40^

Due to its ubiquitous functions, Nrf2 is involved in various diseases. Under normal physiological settings, Nrf2 is inactive and remains in the cytosol. The particularities that Nrf2 displays in diabetes is that the hyperglycemic status is a strong stimulus for oxidative stress and inflammation, and these pathological conditions downregulate the activity of Nrf2 through various neuroinflammatory pathways. Acute hyperglycemia increases Nrf2 expression, but a persistent hyperglycemia decreases its expression. This downregulation of Nrf2 causes various microvascular changes and induces DN therefore distinguishing Nrf2 involvement in DN in comparison with other diseases.^40^

**FIGURE 1:**
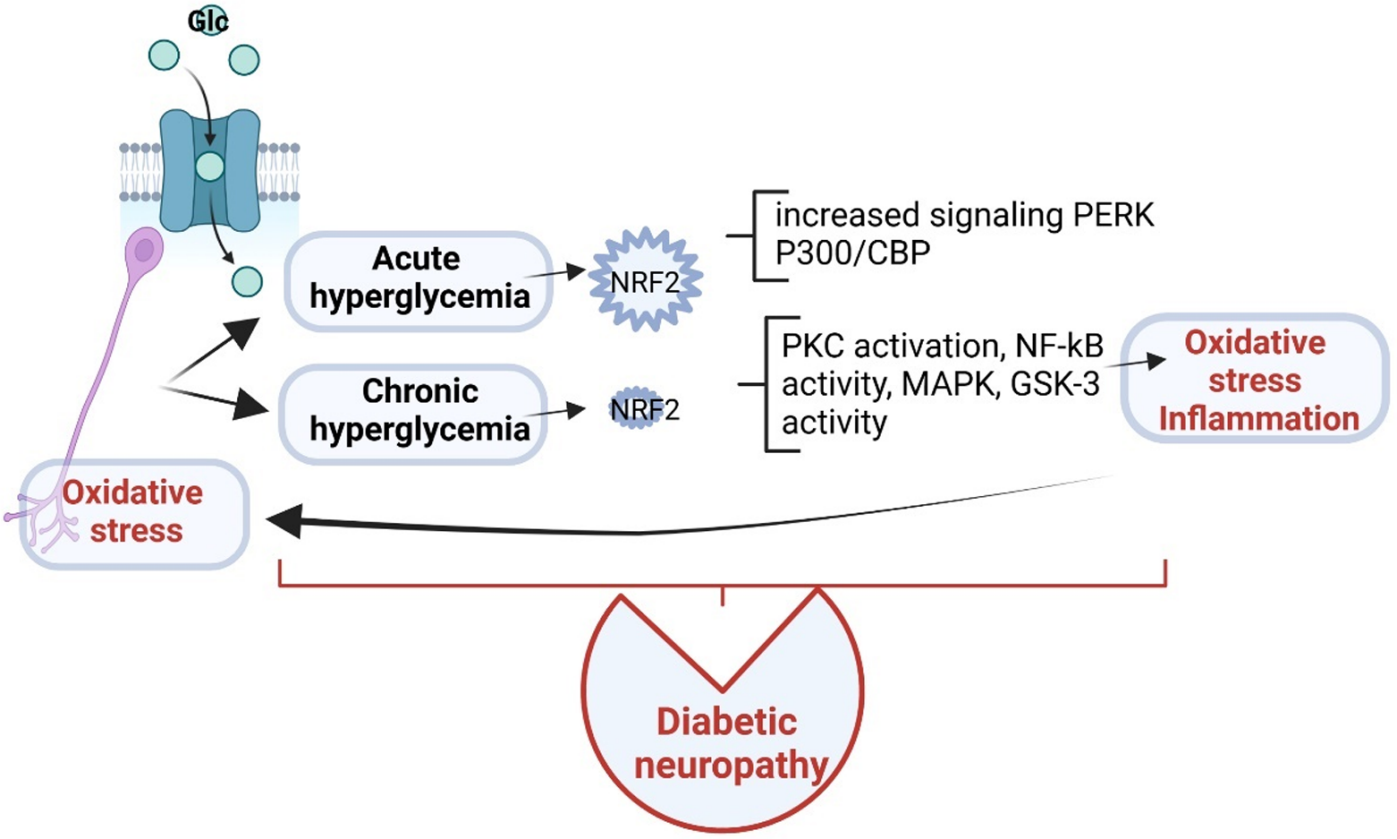
Molecular pathways of NRF2 involvement in DN. Glucose (Glc) intake is increased in diabetes leading to increased oxidative stress at the neuron level. Acute hyperglycemia associates an increase in NRF2 that induces protein kinase RNA‐like endoplasmic reticulum kinase (PERK) and p300/cellular retinol binding (CRB)‐binding protein (P300/CREB) activation. Chronic hyperglycemia induces a decrease in NRF2 that activates protein‐kinase C (PKC), activates NF‐kB, mitogen‐activated protein kinase (MAPK), and glycogen synthase kinase‐3 (GSK‐3). The alteration increases oxidative stress and sustains inflammation contributing to the development of DN. DN, diabetic neuropathy; NF‐κB, nuclear factor‐κB; NRF2, nuclear factor erythroid 2‐related factor 2.

A general scheme of NRF2 pathway involvement is depicted in Figure 1.

NRF2 research is still an evolving field and new roles of the transcription factor are to be discovered, mainly in the pathology of various diseases. We will review some updated aspects related to NRF2 involvement in DN, the animal models that brought important scientific information on this topic and, finally, the interplay between NRF as marker and/or as therapeutical target.

## 2 MOLECULAR EVENTS APPENDING TO NRF2 FUNCTION

### 2.1 NRF2 and noncoding RNA

Noncoding RNAs (ncRNAs) are a group of RNA molecules with heterogenic structures that play a major role in posttranscriptional gene regulation and epigenetic gene silencing.^41^ ncRNAs comprise microRNAs (miRNAs), long noncoding RNAs (lncRNAs), small interfering RNAs (siRNAs), circular RNAs (circRNAs), competing endogenous RNAs (ceRNAs) and piwi‐interacting RNAs (piRNAs).^42^ miRNAs are structurally shaped by the action of two endonucleases, RNase III enzyme drosha and RNase III endonuclease dicer complex. Over 10 years ago it was demonstrated on an experimental model that a knockdown of the dicer enzyme‐encoding gene performed in pancreatic beta cells induces the development of diabetes.^43^ More recently the ncRNA interactome in neuropathic pain was published^44^ and it was revealed that microRNA structure assembly involves NRF2/mitogen‐activated protein kinase (MAPK) signal transduction.^45^ Thoroughly revised by Pandey et al,^46^ the involvement of ncRNA in DN focused on miRNA, lncRNA that is associated with this diabetes complication. Therefore, several years ago upregulated miRNAs were reported in DN^47^ with overexpression of miR‐199a‐3p species. This upregulation induces the inhibition of serine protease inhibitor (SerpinE2) contributing to the tissue vascular damage associated with DN.^48^ LncRNA MALAT1 was found upregulated in the peripheral‐blood mononuclear cells (PBMC) of DN patients.^49^ It is known that MALAT1 can induce neuroinflammation by recruiting EZH2 to the NRF2 promoter and thus impeding Nrf2 expression.^50^ Thus, MALAT1 inhibits NRF2, and induces inflammasome activation and ROS production in neuronal‐based in vitro and in vivo settings.

In DN, downregulated miRNAs were also reported, namely miRNA‐146a was found underexpressed in the PBMC of DN patients. miRNA‐146a downregulation activates the nuclear factor‐κB (NF‐κB) gene that induces the overexpression of tumor necrosis factor‐alpha (TNF‐α) and interleukin‐6 (IL‐6),^51^ potent inflammatory cytokines that are associated once more with DN.^52^

### 2.2 Epigenetic regulation of NRF2/KEAP1

The NRF2‐KEAP1 pathway is epigenetically regulated by genes that can be reversible or heritable during cell division. The epigenetic regulation of KEAP1/NFE2L2 expression is performed through several mechanisms such as DNA methylation, histone modification, and miRNAs, which are epigenetic modulations that can represent new therapeutic targets.^53^ Multiple species of miRNAs have been demonstrated to regulate the NRF2‐KEAP1 pathway. For example, NRF2 overexpression activates the expression of miR‐144‐3p through binding to the AREs in the miR‐144‐3p promoter region. In lung cancer, it was demonstrated that if miR‐144‐3p is targeted, downregulation of NRF2 is induced, subsequently decreasing the expression of genes related to drug resistance.^54^ Concomitantly, in lung tumors downregulation of the miR‐27 family and upregulation of miR‐200 family has a modulatory action on the NRF2/KEAP1 axis.^55^

The role of lncRNA MALAT1 in immune regulation (eg, inflammatory cytokines induction) and angiogenesis is reported in diabetic complications.^56^, ^57^ Recent data has shown that lncRNA MALAT1 has an important role in the antioxidant defense system.^58^ The hyperglycemia status enhances Sp1 binding at the MALAT1 promoter and elevated lncRNA MALAT1, enhances the binding of Sp1 at the Keap1 promoter, and activates its transcription. High Keap1 expression hinders Nrf2 nuclear movement, obstructing the transcription of the antioxidant response enzymes. Inhibiting lncRNA MALAT1 by its siRNA would lead to inhibition of Keap1 upregulation, thus liberating Nrf2 to translocate into the nucleus and to induce the transcription of antioxidant genes, namely the ones that encode HO1 and Sod2. LncRNA MALAT1 is a highly conserved lncRNA and it is linked to various pathologies including diabetes‐related complications.^59^, ^60^ If inhibition of lncRNA MALAT1 is performed, diabetic neurodegeneration is alleviated.^61^ Moreover, lncRNA antisense noncoding RNA inhibits NF‐kB–mediated signaling reducing the overall proinflammatory processes.^62^

## 3 NRF2 REGULATOR OF IMPORTANT CELLULAR PATHWAY

NRF2 as a transcription factor regulates the expression of hundreds of genes involved in the stress response. Discovered almost 30 years ago,^63^ the couple NRF2‐repressor KEAP1 was studied in view of their molecular mechanisms, and their link with various cellular processes within their future clinical application. Thoroughly revised by Kopacz et al there are seminal issues in the NRF2/KEAP1 research domain; solving these issues could bring new therapeutical avenues.^64^

### 3.1 NRF2 and oxidative stress

As already stated, NRF2 regulates antioxidant cellular response; this was its first discovered function, but lately other pathways accompanying cell survival, detoxification, metabolism, autophagy, proteostasis, inflammation, immunity, and differentiation were uncovered. Hence, NRF2 regulates hundreds of genes and is considered to be the hub of a vast regulatory complex system.

**FIGURE 2:**
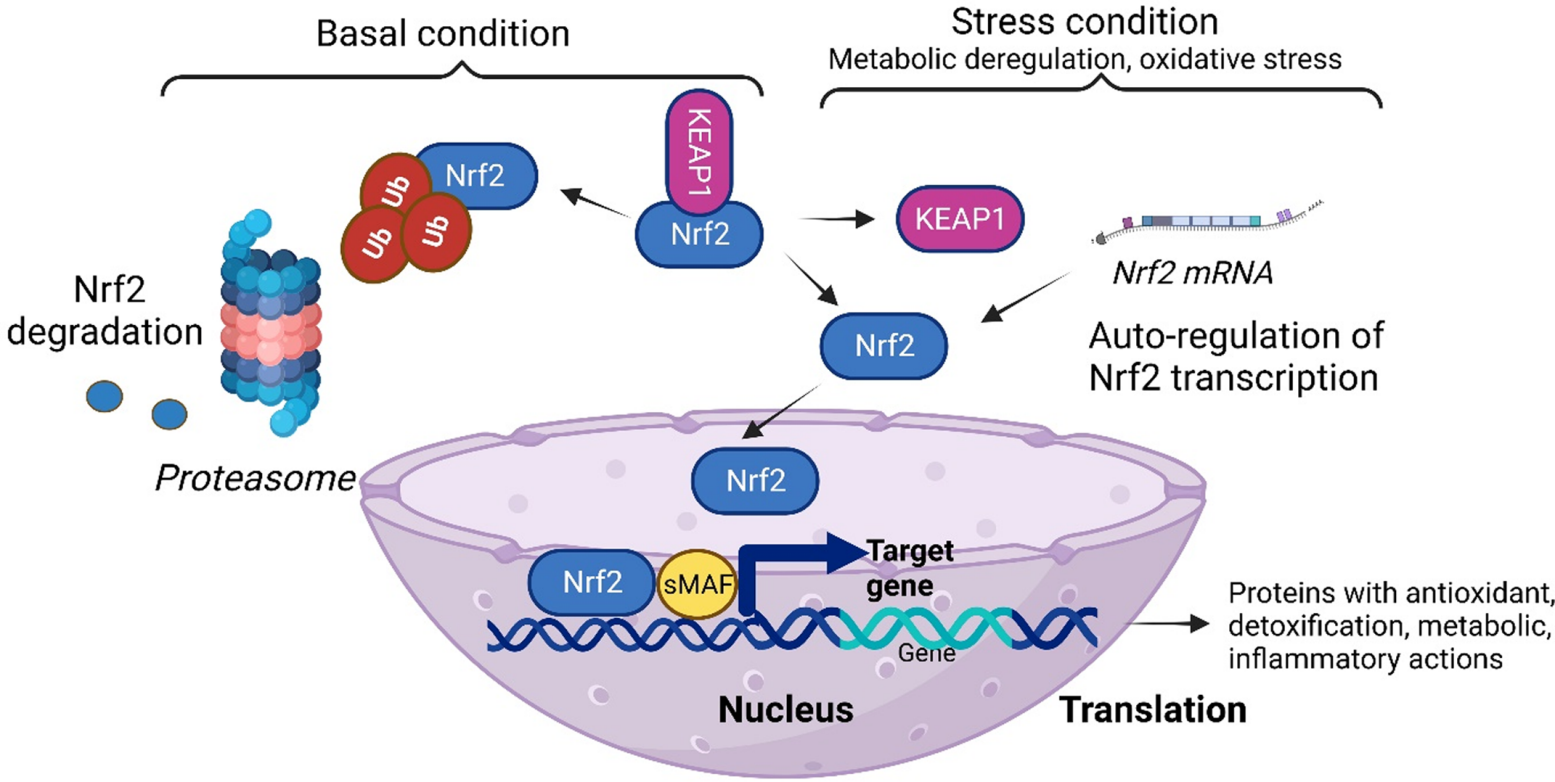
NRF2 molecular pathways. When the cell is under basal conditions, NRF2 levels are low because it is complexed with KEAP1 that leads to proteasomal degradation. When the cell is subjected to various stress conditions like metabolic deregulations, oxidative stress, oncogenic signals, gene mutations, and so on, NRF2 levels increase, because the KEAP1‐NRF2 complex is disrupted. Hence, NRF2 will accumulate in the nucleus, interacting with other transcription factors and cofactors (eg, small musculoaponeurotic fibrosarcoma proteins – sMAF) inducing the transcription of target genes and the translation of proteins with antioxidant, detoxification, metabolic, and inflammatory actions. NRF2 induces an autoregulatory loop inducing its own *NFE2L2* mRNA. KEAP1, Kelch‐like‐ECH‐associated protein 1; NRF2, nuclear factor erythroid 2‐related factor 2; Ub, ubiquitin.

NRF2 regulates the oxidative stress, and the generated ROS are involved in complex physiological/pathological processes starting with normal physiology such as aging, and ending with pathological status such as obesity, cancer, diabetes, and neurodegenerative diseases.^65^ NRF2 regulates more than 50 redox homeostasis‐related genes.^66^ In cellular conditions that are characterized by the absence of ROS, Keap1 links to NRF2 and promotes its proteasomal degradation. This constitutive degradation of NRF2 guarantees that no augmentation of the NRF2 action will occur on target genes. When ROS accumulate, the Keap1‐NRF2 link is disrupted. During oxidative stress, the generated molecules react with sensor cysteines within KEAP1 (eg, cysteine 151, C151, C273, C288) and NRF2 escapes the degradation.^67^ Consequently, the newly synthesized NRF2 builds up in the nucleus activating the cytoprotective genes expression.^3^ Hence NRF2, using its sMAF proteins will bind to ARE in the regulatory regions of genes. Table 1 summarizes the genes that are regulated by NRF2 in the oxidative stress context^68^ and Figure 2 depicts the molecular events^6^ that govern NRF cellular pathways.

**TABLE 1:** NRF2 targets in the redox system.

Molecule (abbreviation)	Action
Thioredoxin	Elimination of ROS
Thioredoxin reductase	
Sulfiredoxin	
Peroxiredoxin	
Glutathione peroxidase	
Superoxide dismutase 1 (SOD1)	
Catalase (CAT)	
Glutathione S‐transferases (GR)	Reduction of glutathione (GSH) for the GSH regeneration NADPH‐mediated
Glutamate cysteine ligase (GCL)	Production of the cellular antioxidant glutathione (GSH)
NAD(P)H quinone oxidoreductase 1 (NQO1)	Detoxification/activation of quinones
Heme oxygenase 1 (HO‐1)	Degradation of heme to produce biliverdin, ferrous ion, carbon monoxide.

Abbreviation: ROS, reactive oxygen species.

NRF2 regulates antioxidant pathways inducing enzymes to produce reduced glutathione (GSH) consumption and regeneration. For the GSH synthesis NRF2 will regulate glutamate‐cysteine ligase catalytic and modulator subunits and glutathione synthetase. Besides the redox enzymes presented in Table 1, glutathione reductase uses NADPH, a cofactor from anabolic reactions.^69^

In the landscape of redox complex systems, NRF2 has several targets^6^ (Table 1).

In the context of oxidative stress, NRF2‐target genes encode for cytoprotective proteins against toxic and oxidative injuries. Therefore, oxidative stress in pathological conditions can be identified in various diseases such as cardiovascular, metabolic, neuronal degeneration, autoimmune disorders, and cancer.^6^, ^65^, ^70^, ^71^, ^72^

### 3.2 NRF2 and metabolic pathways

NRF2 regulates almost 200 genes influencing metabolism and cellular repair.^73^ In the context of DN, NRF2 action becomes extremely important in the metabolic area. NRF2 is involved in various layers of metabolic functions. It regulates mitochondrial functions, nutrients uptake, anabolism, macromolecular biosynthesis, and energy metabolism. All these metabolic layers sustain the complex cell physiology. Using chromatin immunoprecipitation sequencing (ChIP‐Seq) analysis in cell lines^74^ or in animal models,^75^ numerous metabolic‐related genes were revealed as NRF2 targets. Genes involved in glycolysis regulation, pentose phosphate pathway, fatty acid pathways, glutamine, and glutathione metabolism were identified as NRF2 targets. In the brain, NRF2 expression is increased in astrocytes and microglia compared to neurons.^76^ It is highly probable that the information circulation between astrocytes and neurons requires an increased metabolism so that astrocytes provide neurons with glycine, glutamate/glutamine and cysteine, namely GSH precursors supporting neuron function.^77^

In animal models of diabetic db/db mice that have Keap1 gene knockdown (Keap1flox/−) it was shown that NRF2 activation inhibits gluconeogenesis by suppressing several molecular targets (eg, glucose‐6‐phosphatase, fructose‐1,6‐bisphosphatase 1, phosphoenolpyruvate carboxykinase [PCK1], peroxisome proliferator‐activated receptor g coactivator 1‐a, nuclear receptor subfamily 4, group A, member 2), preventing the initiation of diabetes.^78^ Liver‐specific constitutive NRF2 activation induces the reduction of gluconeogenesis through PCK1 and pyruvate dehydrogenase kinase genes.^6^

NRF2 is involved in various stages of the glycolysis cycle. Glucose‐6‐phosphate is converted by phosphoglucomutase (PGM) to glucose‐1‐phosphate. Within the glycogen metabolism cycle NRF2 activates PGM5, 1,4‐alpha‐glucan branching enzyme 1, phosphorylase kinase regulatory subunit alpha 1, and glucosidase alpha acid. The liver and the skeletal muscle are the main sites for glycogen synthesis/storage, and NRF2 regulates these processes.^79^ In mouse models it was shown that constitutive NRF2 activation liver induces glycogen accumulation maintaining blood glucose during fasting.^79^ Meanwhile, in animal models of skeletal muscle‐specific Keap1‐knockout mice, a decreased glycogen content is identified in the skeletal muscle, along with an improved glucose tolerance.^79^

The metabolic pathways of iron and oxygen are functionally linked as iron is an essential cofactor element of enzymes involved in the major cellular chains: oxygen transport, oxidative phosphorylation, and metabolite oxidation. NRF2 regulates several genes that further regulate the iron metabolism. Recent studies have shown that genes involved in heme synthesis, hemoglobin catabolism, iron storage, and iron export are controlled by NRF2. Besides cancer, NRF2 activation and iron metabolism deregulation^80^ were also noted in diabetes. Ten years ago, it was mentioned that the pathophysiology of diabetes revealed that iron accumulation sustains an increased risk of type 1 and type 2 diabetes.^81^ As metabolic stress involves complex processes, one of these is the imbalance of the redox equilibrium that induces tissue damage and pancreatic β‐cell death. These cells are vulnerable to oxidative damage due to the iron accumulation and β‐cell ferroptotic‐like deregulation. Recent studies have shown a link between iron metabolism and redox status in diabetes based on ferroptotic‐like deregulation of pancreatic β‐cells.^82^ Ferroptosis (a type of iron‐dependent and nonapoptotic cell death) was identified in the pancreatic β‐cell pathophysiology. The accumulation of toxic ROS and the deregulated ion metabolism^83^ are both linked and controlled in diabetes by NRF2.

### 3.3 NRF2 in inflammation and immunity

Inflammation is a complex process having various roles, including the elimination of numerous aggressors, activating innate and adaptive immune cells. Many regulatory mechanisms control this inflammatory network, so that the processes resume after the pathogenic insult is eliminated and cell/tissue regeneration occurs. Bypassing these control mechanisms will lead to the chronic form of inflammation. Chronic inflammation is the trigger for many diseases from autoimmune diseases and neurodegenerative diseases to cancer.^84^ Regulation of inflammation is performed through toll‐like receptor (TLR) pathways and is NRF2 mediated. TLR pathways controlled by NRF2 establish a connection between immune regulation and the oxidative stress responses via inflammation regulation. This close TLR‐NRF2 link in the inflammatory context is to be found as the basis of many diseases. Targeting this molecular “couple” can lead to the development of future therapeutic strategies.^85^

Autoimmune disorders are known to have an excess of ROS generation, proven in the large array of autoimmune diseases from psoriasis to arthritis and from diabetes to Crohn's disease. Other conditions like obesity and aging add immune dysfunction and oxidative stress to these autoimmune deregulations. The accumulation of ROS triggers the activation of inflammatory cells that further sustain the inflammatory status through the secretion of inflammatory cytokines, chemokines, and factors that mediate various signal transduction and transcription pathways (eg, NF‐κB, signal transducer and activator of transcription 3, hypoxia‐inducible factor‐1 alpha, NRF2). The antioxidant enzymes (SOD and catalase) are imbalanced; therefore, therapies that supplement this deregulation of the defensive natural component can alleviate various autoimmune pathologies.^86^

The expression of NRF2 in immune cells and in circulating blood cells was reported almost 20 years ago.^63^ The highest levels of NRF2 were found in circulating monocytes, neutrophils, and T and B cells, which suggests a clear control of this transcription factor in the physiology of immune cells.^87^ Any injury, infection, or chemical/physical trigger will initiate an inflammatory reaction where innate immune cells will be the first line of cells that will arrive at the injured site. Inflammatory cells will eliminate the infectious agent and will initiate the tissue repair processes.^84^ ROS are an extended family of signaling molecules and mediators of inflammation.^6^ As already stated, chronic inflammation develops as an uncontrolled process that leads to cell damage and cellular proliferation. Alongside many diseases, chronic inflammation is a stepping stone to diabetes.^77^ NRF2 modulates redox metabolism alleviating ROS by directly inhibiting proinflammatory cytokine genes or inflammatory NF‐κB signaling.^6^ Using ChIP‐seq that analyzes protein–DNA interactions, it was demonstrated that NRF2 binds directly to the promoter regions of IL‐6 and IL‐1β genes. This binding hinders RNA polymerase II enlistment blocking gene induction in innate immune cells, namely in macrophages.^88^ NRF2 hinders IL‐6 expression by ARE‐dependent induction suppressing IL‐6 transcription.^89^ NRF2 has a modulatory action by suppressing or activating immunity depending on the cellular physiology. For example, NRF2 activation within T cells lowers T activation and differentiation.^90^

### 3.4 In vitro and in vivo models exploring NRF2 molecular events in DN

Animal models have been widely used to study the molecular mechanisms that are controlled by NRF2. In a *Caenorhabditis elegans* model, the aging processes and related pathologies, including neurological complications of diabetes were studied. Hence α‐dicarbonyls (α‐DCs) accumulate with age and, in mutated animals (glod‐4), hyperesthesia and neuronal damage was shown. TRPA‐1 acts as a sensor for α‐DCs, that has a conserved structure from worms to mammals. Furthermore, TRPA‐1 stimulates SKN‐1/NRF through calcium‐regulated kinase signaling, so that the detoxifying of α‐DCs is activated. Chaudhuri et al have shown in an animal model that TRPA‐1 can be a drug target for diabetes and neurodegenerative diseases.^91^

Most animal models developed in this area are rodent models and several mechanisms were studied using these models. Chronic inflammatory status governs major diseases, therefore drugs that alleviate the inflammation are constantly being developed. These drugs are characterized by side effects, hence discovering other treatment approaches in the fight against chronic inflammation is a constant goal of the last decades. Natural‐derived molecules are good candidates in these studies. Recent reports have shown Withaferin A (WA), a bioactive molecule isolated from *Withania somnifera*, as having an antichronic inflammatory action. WA triggers NF‐kB and NRF2 signaling pathways and has proven its efficacy in both in vitro and in vivo animal models of various diseases including diabetes and neurodegenerative disorders.^92^

Another natural polyphenolic derivative of benzoic acid, syringic acid (Syr) was tested in animal models to alleviate the oxidative stress status in diabetes and to improve the complications of DN. Syr was proved to eliminate ROS and modulate various transcriptional factors, including NRF2 in diabetic animal models.^93^ Furthermore, Zhao et al found that streptozotocin (STZ)‐induced DN is correlated with antioxidant deficiency in the spinal dorsal root ganglia and the sciatic nerve.^94^ More recent data^95^ have shown that malondialdehyde (MDA) level in the brain, sciatic nerve, and spinal cord significantly increase in diabetic animals, and that Syr administration reduces MDA level in tissues proving Syr's antioxidant effect. Oxidative stress and impairment of antioxidants contribute to the damage of cognitive functions.^96^, ^97^ Ogut et al have shown in animal models that Syr can improve learning dysfunction and memory suppression through oxidative stress reduction.^98^ Other natural compounds like rosmarinic acid,^99^, ^100^, ^101^ epigallocatechin‐3‐gallate, gallic acid,^102^ and ellagic acid^103^ were documented as improving memory impairment. This memory impairment due to hyperglycemia is a consequence of cholinergic dysfunction, and therapies that influence cholinesterase activity are to be considered as important in diabetes‐induced neurodegeneration.^104^ Some phenolic acids (eg, chlorogenic acid) in animal models reduced acetylcholinesterase in the hippocampi of cognitive deficits animals.^105^ Around 20 years ago it was still unclear whether mitochondrial biogenesis has a neuroprotective mechanism in DN.^106^ More recent studies have shown that mitochondrial biogenesis promotes cellular viability.^107^ Additionally, in neurons, oxidative stress induces mtDNA injury, corroborated with reduction of mitochondrial biogenesis leading to the development of DN.^108^ Contrarywise, processes leading to mitochondrial regeneration/biogenesis could decrease the risk of DN.^109^ Hence, Syr administration induced an increase in the copy number of mitochondria in the brain and spinal cord.^93^ At the genetic level, the process increases gene expression for peroxisome proliferator‐activated receptor‐γ coactivator 1 alpha (PGC‐1α), mitochondrial transcription factor A (TFAM), NRF1 and NRF2, and mitochondrial transcription factor B1.^110^, ^111^ Indeed, Syr administration increases the expression of PGC‐1α and NRF‐2 mRNA in the brain, inducing mitochondrial biogenesis. Hyperglycemia alters PGC‐1α activator, which consequently leads to TFAM and NRF2 expression impairment and mitochondrial dysfunction.^112^ Various neurodegenerative diseases (eg, Alzheimer's, Parkinson's, amyotrophic lateral sclerosis, and Huntington's disease) demonstrate these molecular deregulations,^112^ whereas in diabetes, a decrease of PGC‐1α and NRF‐1 gene expression was found.^113^ In PGC‐1α‐knockout mice, experimentally triggered diabetes led to more severe DN.^114^ It was reported that, in physiological conditions, the blood‐nerve barrier hinders the entrance of circulating T lymphocytes, whereas hyperglycemia damages this nervous‐vascular barrier and favors their entrance. Glycosylated myelin can be an antigen for the immune cells and will be subjected to phagocytosis by the innate immune cells.^115^ Inflammatory cytokines, TNF‐α and IL‐1β, were proven to have deleterious effects on neurons and glial cells, and the induced neuroinflammation will lead to demyelination.^115^ Modulating these inflammatory cytokines, namely reducing TNF‐α, IL‐1β, and IL‐6 secretion, would lead to a decrease in the sciatic nerve demyelination.^116^

In another rat model of diabetes, vitamin D and rosuvastatin were studied for their neurotherapeutic potential. Various parameters were investigated in this animal model, from small/large nerve electrophysiological examination to neuronal inflammation, apoptosis, and various intracellular pathways. This animal model showed that vitamin D and/or rosuvastatin improved diabetes‐induced neuropathy through suppressing Notch1 and Wnt‐10α/β‐catenin pathways that hindered neuronal degeneration.^117^ Rutin (a ubiquitous plant flavonoid) was also studied in animal models of DN. Pain assessment, motor nerve conduction velocity, and complex oxidative parameters were assessed in this animal model subjected to rutin therapy. Treatment with rutin and nimesulide (nonsteroidal anti‐inflammatory drug) attenuated all neurological parameters, with the combination having the highest effect on experimental DN.^118^

In vitro studies were performed on cultured dorsal root ganglion (DRG) neurons in order to assess various therapeutical compounds. High glucose levels noticeably increased DRG apoptosis by increasing intracellular ROS and activating the NF‐κB signaling pathway. If cells were subjected to quercetin (Q), cinnamaldehyde (C), and hirudin (H), then caspase‐3 activation and apoptosis were significantly reduced. The expression of various molecules decreased after in vitro therapy (NF‐κB, IL‐6, TNF‐α), and NRF2 expression increased. The combination of quercetin, cinnamaldehyde, and hirudin induced the scavenging of ROS, activated NRF‐2 and alleviated inflammatory components.^119^, ^120^ Likewise, in a high‐glucose DRG neurons model it was shown that cells are sensitive to oxidative stress. In an in vitro model, the antioxidant/antiapoptotic effects of calcitonin gene‐related peptide (CGRP) were studied. The report shows CGRP increased the expression of NRF2 and heme oxygenase‐1 (HO‐1), via PI3K/AKT pathway, reducing apoptosis and oxidative stress.^121^

## 4 NRF2 AS DN DISEASE MARKER AND THERAPY TARGET

As shown previously, NRF2/Keap1 pathway incorporates both metabolic and cytoprotective functions that are deregulated in diabetic conditions.^122^ In chronic hyperglycemia, high superoxide anions escape from the electron transport chain within the mitochondria during aerobic respiration^123^ and scavenging potency is overridden so that the oxidation processes at cellular levels are enhanced.^124^ Soares et al have shown several years ago that diabetic skin has low expression of NRF2 and low antioxidant potency that leads to decreased wound healing.^125^ Bone marrow–derived multipotent stromal cells (BMSCs) have a low expression of cytoprotective NRF2/Keap1 in diabetes. If NRF2‐directed transcription is activated, the redox homeostasis is restored in BMSCs and therefore their regenerative potency is recovered.^126^, ^127^

As previously mentioned, NRF2 is studied as a therapy target. Recent studies have shown that astragaloside II (AS II), a saponin purified from *Astragalus spp*, can modulate the immune response and tissue regeneration and stop the inflammatory response. AS II was studied in a STZ‐induced DN animal model evaluating its effect on podocyte injury and mitochondrial dysfunction. AS II ameliorated podocyte physiology and apoptosis in DN rats. Furthermore, AS II increased NRF2 expression and decreased Keap1, enabling antioxidative stress. These results have shown that AS II regulates NRF2 and therefore ameliorates podocyte injury in experimental DN.^128^

The therapeutic effect of bergenin was reported in 2020 in another animal model of STZ‐induced DN. Using the analysis of cytokines, antioxidant genes, oxidative stress markers, nitric oxide production and MDA/nitrite tests, the bergenin effect on DN symptomatology was evaluated. Bergenin proved to have antioxidant properties modulating gene expression, namely downregulating inducible nitric oxide synthase and upregulating glutathione peroxidase and NRF2 in the nervous system. Moreover, bergenin modulated the pro‐ and anti‐inflammatory cytokines in DN mice. After treatment, the cytokines were predominantly anti‐inflammatory and an upregulation of antioxidant pathways was recorded.^129^

In 2022, a set of new cinnamaldehyde compounds were evaluated in vitro, ex vivo, and in vivo for the capacity to restore peripheral nerve degeneration. Between them, the highest potency was demonstrated by a compound having a naphthaldehyde group. Its hydrophobic interactions with transient receptor potential cation channel subfamily A member 1 had the highest binding affinity. This compound hindered the oxidative stress in Schwann cells alleviating neurodegenerative processes.^130^ Trichostatin A (TSA) is a reversible inhibitor of class I and II histone deacetylases, regulating peripheral neurodegeneration. TSA was recently shown to inhibit overall peripheral neurodegeneration (eg, myelin fragmentation, axonal degradation, trans‐dedifferentiation/proliferation of Schwann cells).^131^ Moreover, additional studies have shown that the effect of TSA is associated with the Keap1‐NRF2 pathway.^132^

In a high‐glucose rat model, the effect of hirudin on oxidative stress/apoptosis of spinal dorsal root ganglion cells was studied. This recent report outlines that the activity of spinal dorsal root ganglion cells is supported by hirudin due to its ROS scavenging potency, NRF‐2/HO‐1 pathway up‐regulation, NF‐κ B pathway inhibition, and caspase‐3/apoptosis downregulation.^133^ In another DN rat model published in 2023, the translocator protein agonist Ro5‐4864 was proven to alleviate neuropathic pain. Besides increased myelin sheath thickness and protein expression, the compound improved the expression of NRF2, cytoplasmic HO‐1, and NQO1.^134^ The bioflavonoid morin has a scavenging effect on ROS and it also proved its efficacy in DN animal models. Administration of morin reduced hyperalgesia and allodynia ameliorating mitochondrial SOD, membrane depolarization, and total ROS. The mechanism that morin triggered is based on the increased NRF2‐antioxidant defenses.^135^

Gangliosides are highly involved in neurological diseases and their administration showed beneficial effects since the 80's.^136^ More recently, the mechanisms of mono‐sialo‐tetrahexosyl‐ganglioside ganglioside in the treatment of DN were studied. Actually, this ganglioside attenuates neuroinflammation.^137^ At least in Alzheimer's disease, one of the mechanisms of ganglioside action is through the NRF‐2/ARE signaling pathway.^138^ Lipoic acid is a dithiol compound that reduces ROS, chelates metal ions, and regenerates vitamin C, vitamin E, and glutathione. Through its action, it enhances the antioxidant system, a process that was reported as relying on NRF‐2‐gene expression. Therefore, lipoic acid is another NRF2‐mediated compound that is beneficial in DN, and these results were reported in several clinical trials of DN patients.^139^ Several phytochemicals were tested for their antioxidant potency and their neuroregenerative action. Polyphenols are known to interact with ROS and apigenin has proven its neuroprotective effect against oxidative stress through extracellular signal‐regulated kinase‐independent processes.^140^ Sinomenine is a known alkaloid with anti‐inflammatory, immunosuppressive, and neuroregeneration capacities.^141^ Sinomenine induces mechanisms that rely on mediating ferroptosis of hippocampal neurons. The actions of sinomenine were studied on cellular models of HT‐22 cells and in DN animal models. Sinomenine reduced hippocampus neuronal ferroptosis through the enhancement of EGF expression that activated the NRF2/HO‐1 signaling pathway. The in vitro results were confirmed in the DN animal model.^142^

NRF2 is a marker that indicates whether the cell functions normally or has a profoundly deregulated cellular physiology. NRF2 is similarly a therapy target, as its upregulation can balance the pathological state induced by various factors, hyperglycemia included.

In Table 2 we present the expression of Nrf2 in different diseases including DN and the drugs that target NRF2.

**TABLE 2:** Expression of NRF2 in various diseases and updated drugs that aim to regulate NRF2.

Pathology	Nrf2 status	Action	Drug targeting Nrf2	Drug action	Ref
Prostate cancer and castration‐resistant prostate cancer	Suppressed	Increased autophagy, apoptosis, and necrotic ptosis, favoring tumor metastasis and drug resistance	Neoadjuvant endocrine/chemo therapy	Drugs activate Nrf‐2, which induces the p120‐Nrf1 nuclear accumulation, significantly inhibiting NF‐κB DNA‐binding activity by decreasing the transactivation of androgen receptor	143
Acute myeloid leukemia	Overexpressed inducing autophagy, cytokines, and kinases' expression.	Nrf2 target genes (eg, NADPH quinone oxidoreductase I, glutamate‐cysteine ligase catalytic subunit, glutamate‐cysteine ligase modifier subunit) have NF‐κB binding site and hence these two signaling pathways cooperate in promoting chemoresistance to bortezomib treatment			144
Skin pathology induced by UVB irradiation	Nrf2 degraded by UVB	Nrf2 has a photo‐protective effect through the suppression of MMPs expression			145
Neuronal inflammation	Suppressed	Nrf2 suppression favors oxidative stress and inflammation through the activation of NOX4/ROS/NF‐kB pathway			146
Animal model of subdued to spinal cord injury Nrf2 deficient	Suppressed	Nrf2 suppression induces significant elevation in NF‐κB activation, TNF‐α production, and expression of MMP9			147
Different in vitro, in vivo models and humans with Huntington disease	Suppressed	Nrf2 suppression induces neuroinflammation processes in microglia and macrophages	Triazole derivatives	Nrf2 induction inhibits proinflammatory cytokines	148, 149
Multiple sclerosis	Suppressed	Nrf2 suppression induces low control of antioxidative pathways	Dimethyl fumarate, approved by US Food and Drug Administration	Induces anti‐inflammatory action via Nrf2 antioxidant pathway	150, 151
Diabetes‐associated gastric pathology	Suppressed	Nrf2 suppression reduces the expression of HMOX1 gene with anti‐inflammatory activity			152
Obesity‐induced diabetes	Suppressed	Nrf2 suppression lowers the control on insulin secretion and insulin resistance, inducing hyperglycemia			78
Diabetic skin ulcers	Suppressed	Nrf2 suppression and/or knockdown delays wound healing, increases oxidative DNA damage, reduced TGF‐β1 levels, high MMP9 expression and increased apoptosis			153
Diabetic nephropathy	Suppressed	Increased oxidative damage and inflammation	Epigallocatechin gallate	Induces activation of Nrf2, ERK and p38 MAPK signaling pathways for anti‐inflammation action	154
Neuropathic inflammation	Suppressed	Increased oxidative damage and inflammation	Rosmarinic acid	Induces Nrf2 activation and attenuates chronic constriction injury –induced neuropathic inflammation	155

Abbreviations: MMP, matrix metalloproteinase; NF‐κB, nuclear factor‐κB; NRF2, nuclear factor erythroid 2‐related factor 2; ROS, reactive oxygen species; TGF‐β1, transforming growth factor‐beta1; TNF‐α, tumor necrosis factor‐alpha; UVB, ultraviolet B.

## 5 NRF2 DETRIMENTAL IN HUMAN PATHOLOGY

As already stated, Nrf2 is located at the intersection of crucial signaling pathways, regulating various cellular functions, beyond the redox balance, and is involved in cellular metabolism, proteostasis, mitochondrial function, and inflammation. However, due to its complex involvement, the specific time frame for NRF2 activation or inhibition is of seminal importance. NRF2 signaling has an intrinsic dualism mediating beneficial effects but also some detrimental effects in biological processes associated with aging, neurodegeneration, metabolic diseases, long‐COVID‐19, and carcinogenesis.^156^ For example, Nrf2 has a context‐dependent manner of action in the cardiovascular system. Recent emerging data show the unexpected role of Nrf2 in mediating cardiovascular maladaptive remodeling and dysfunction in vascular diseases. All these findings gain clinical relevance having Nrf2 pathways as targets in cardiovascular diseases.^157^ In bacterial infections, a recent report has shown another detrimental effect attributed to Nrf2. Besides various roles, immunity maintains the host's iron homeostasis sequestrating iron from pathogens and hence controlling the infection. Nucleotide‐oligomerization domain‐like receptors (NLRs) are highly involved in this process. Moreover, it was shown that NLRP6 alters the host resistance to bacterial infection. NLRP6 deficiency upsurges the iron exporter ferroportin‐mediated iron efflux through a NRF2‐dependent process. Therefore, NLRP6 becomes a therapeutic target for limiting bacterial iron acquisition.^158^

As mentioned before, increased NRF2 activity is beneficial for proper health, nevertheless, the fact that NRF2 becomes activated in neoplastic transformed cells is detrimental. Tumor cells display persistent NRF2 activation that govern their therapeutic resistance and aggressiveness. This persistent activation of NRF2 confers molecular advantages for these transformed cells in terms of genetic, metabolic, and immunological characteristics underlaying the detrimental action of NRF2 in tumorigenesis.^159^

Recent cumulative evidence points out that Nrf2 activators and their involvement in human pathology should be kept in mind before introducing them in clinical practice.

## 6 CONCLUSION

NRF2 is one of the recent leading subjects of extensive research. Studies regarding NRF2 concentrate upon inflammation, metabolism, and oxidative stress as foremost factors in human pathologies. First identified as an oxidant stress response transcription factor, recent studies have shown its ubiquitous involvement in regulation of seminal cellular processes such as metabolism, inflammation, autophagy, proteostasis, mitochondrial physiology, and last but not least immune responses. These key regulator functions of NRF2 are sustained by the fact that this transcription factor is centered in a complex regulatory network that controls important functions. Additionally, due to its central role, NRF2's interconnections contribute to the initiation and development of metabolism‐ or inflammation‐associated diseases, diabetes included.

As diabetes and prediabetes are alarmingly increasing worldwide, and knowing that half of the patients will sustain injuries of the peripheral and autonomic nervous systems, developing various forms of DN, the studies of the intimate molecular mechanisms are seminal. The pathophysiology of DN involves inflammatory and apoptotic complex pathways. which are bridged by NRF2. Through its ARE binding site, NRF2 regulates antioxidant proteins (eg, detoxifying enzymes). Moreover, NRF2 links neuroinflammation and apoptotic pathways affecting the progression of DN.

There are several challenges regarding the current research in Nrf2. Therefore, when evaluating the pharmacodynamic action of NRF2 inducers, genetic and biochemical biomarkers should be evaluated. For example, the Nrf2 induction has to evaluate NQO1 transcript, epigenetic regulators like histone deacetylase, oxidative stress markers, pro‐ and anti‐inflammatory cytokines, NF‐κB signaling molecules and many other indicators of a proper functioning of induced Nrf2. Moreover, the action of inducers should be closely followed in a dose–response manner and standardized methodologies should be used to validate the obtained results. In the testing workflow, another general issue is that animal models do not exactly reproduce human pathology. Therefore, animal models that best recapitulate human diseases in terms of Nrf2‐related pathogenic mechanisms are to be obtained in the future. In these preclinical studies, new Nrf2 modulators can be tested. It is well known that noncommunicable diseases are characterized by multiple pathways and high complexity. As Nrf2 is involved in various pathologies, its mechanism can be similar or different. Therefore, the Nrf2‐intimate molecular mechanisms that lead to a specific disease should be thoroughly analyzed to identify new Nrf2 modulators or even to repurpose existing drugs. Deciphering the molecular mechanisms of Nrf2 is also important for the evaluation of additional networks. These networks can be disturbed when triggering Nrf2, so any potential unwanted side effects of a drug can occur and hence should be foreseen. Another issue that requires attention is Nrf2's partner, Keap1, which should also be investigated in order to detect structural subtleties as well as high‐ and low‐affinity active sites that could hinder/modulate the Keap1 interaction with Nrf2. When using tentative drugs that aim at active residues, these drugs can also affect other proteins, hence the importance to thoroughly study the structural subtleties should be highlighted. Another issue that challenges the Nrf2 domain is that Nrf2 activators cannot cross the blood–brain barrier in neurological disorders.^160^ Furthermore, the positive results of Nrf2 targeted drugs obtained in preclinical settings still need to be validated in clinical trials.

Future studies will have to focus on the mechanisms that can be modulated at the mitochondrial genes, mitochondrial biogenesis, and ATP content of brain and spinal cord tissues to find new therapeutic properties of drugs to be used in DN. All these studies can have NRF2 activators as a starting point to alleviate the progression of DN through NRF2‐mediated mechanisms.

Without being exhaustive, our paper focused on the recent published data regarding the contribution of NRF2 in the installment and progression of DN.

## AUTHOR CONTRIBUTIONS

Monica Neagu, Carolina Constantin, Constantin Caruntu: Literature search, data collection, original draft preparation; Mihaela Surcel, Adriana Munteanu, and Monica Neagu: Writing—review and editing. Monica Neagu, Carolina Constantin, Constantin Caruntu, Cristian Scheau, and Ilinca Savulescu‐Fiedler: conceptualization, methodology, supervision, project administration, writing—review and editing. All authors have read and agreed to the published version of the manuscript.

## FUNDING INFORMATION

The work and open access fee was supported by a grant of the Romanian Ministry of Research, Innovation and Digitization, CCCDI‐UEFISCDI, project number [PN‐III‐P2‐2.1‐PED‐2021‐2243 and 31PFE/30.12.2021], within PNCDI III.

## DISCLOSURE

The authors declare no conflict of interest.

## INSTITUTIONAL REVIEW BOARD STATEMENT

Considering that only previously published papers were used for the systematic review, there are no ethical concerns with this study. The institutional review board confirmed that no ethical approval was needed.

## INFORMED CONSENT STATEMENT

Not applicable.

## Data Availability

The data presented in this study are available on request from the corresponding author.
